# Characterizing Nigerien men's social networks and their influence on family planning-related attitudes and behaviors

**DOI:** 10.1016/j.ssmph.2022.101203

**Published:** 2022-08-16

**Authors:** Sneha Challa, Nicole Johns, Abdoul Moumouni Nouhou, Ricardo Vera-Monroy, Jay G. Silverman, Holly Shakya

**Affiliations:** a Center on Gender Equity and Health, University of California San Diego, 9500 Gilman Dr, Mail Code 0507, La Jolla, CA, 92093, USA; b The OASIS Initiative – Niger, Oasis Initiative, Niamey, Niger

**Keywords:** Social networks, Social norms, Family planning, Adolescents, Niger

## Abstract

This work uses data from a family planning (FP) program evaluation and social network study among men married to adolescent girls (ages 13–19) in Dosso, Niger to explore who influences their FP and through which social mechanisms. We asked men (N = 237) to nominate and describe their perceptions of key social contacts (alters). We sought to interview the most influential alter (N = 157 interviewed alters), asking them about their own FP-related attitudes and behaviors. Men primarily nominated male friends as alters. We found that men participating in the program were more likely to perceive alters to hold attitudes supportive of gender equitable FP decisions (AOR: 4.36, 95% CI: 1.83, 10.35) and FP use (AOR: 4.22, 95% CI: 1.72, 10.35). Alters' attitudes supporting FP were related to those of the men who nominated them (1-unit increase in alters' attitudes score related to a 0.48 unit increase in men's attitudes; 95% CI: 0.32, 0.63). Men who perceived their alters would support gender equitable FP decisions were more likely to have ever used FP methods (AOR: 10.43, 95% CI: 2.50, 43.58) as were those who perceived their alters would support their own FP use (AOR: 12.76, 95% CI: 2.55, 63.81). Men who perceived their alters would support gender equitable FP decisions were more likely to report spousal communication (AOR: 8.71, 95% CI: 3.06, 24.83), as were those who perceived that alters would support their own FP use (AOR: 9.06, 95% CI: 3.01, 27.26). Alters' and men's behaviors (contraceptive use and spousal communication) were not associated. These results demonstrate that perceived approval from network members may be critical to FP-related attitudes and behaviors. However, since FP promotion programs may affect perception and/or composition of social networks, future research should include larger sample sizes and longitudinal data to understand the effect of changing norms on social relationships.

## Highlights

• We collected social networks data from 237 men in the Dosso District of Niger.
• Nigerian men's social networks comprise primarily men friends.
• Men's FP attitudes were associated with their perceptions of network members.
• Men's FP-related behaviors were not associated with those of their network members.

## 1 Introduction

Niger, with a population of roughly 23 million people, has the highest total fertility rate in the world (over 7.0 births per woman) (United Nations, 2015). A major influence on the fertility rate is the high prevalence of early marriage, with three-fourths of girls married by age 18 years (United Nations Children's Fund, 2017). Ethnographic work has demonstrated that subsequent to marriage, adolescent wives face pressure to bear children as a way to prove their fertility and fulfill the responsibility it is believed God intended for them (Samandari et al., 2019). In this context, where gender segregation is the norm and men are seen as the heads of household, adolescent wives' decision-making power and ability to engage in couple communication are diminished. (Masquelier, 2005; Perlman & Chaibou, 2018) Given this division of power and labor, decision-making around family planning (FP) remains in men's hands as the key facilitators of FP behaviors (Family Planning 2020, 2018; Perlman & Chaibou, 2018). As such, engaging men may be key to increasing the acceptability of FP-related behaviors and supporting women who desire limiting or spacing their births to achieve their fertility goals. The results of Husbands Schools, a program that involved dialogue around social norms related to fertility between men who held gender equitable attitudes their peers with less gender equitable attitudes, demonstrated that peer support and communication among men has had a positive effect on women's access to healthcare (United Nations Population Fund (US), 2011). Thus, an improved understanding of how relationships within peer groups influence men's attitudes and behaviors may be key to uncovering more about the context surrounding FP decision-making processes and engaging men as supportive partners. To this end, FP programs have increasingly sought to involve male partners.

Simultaneously, there has been increasing recognition that favorable health outcomes are defined by individual needs and preferences which in turn, are informed by the social context of each individual. (Price & Hawkins, 2007) Thus, more public health research has begun to include ecological approaches and analysis of social networks data, allowing for consideration of the interpersonal and broader social environment (Green et al., 1996; Luke, 2005; Luke & Harris, 2007). Social network data and analysis offer a lens through which to view how information, ideas, and behaviors spread through social mechanisms. A conceptual model presented by Berkman et al. identifies four major social mechanisms: 1) social support, 2) social engagement, 3) access to resources, and 4) social influence (Berkman et al., 2000). A fifth potential mechanism is social learning, and as evidence from social network studies has grown in the SRH sphere, it has pointed to social influence (adoption of an attitude/behavior because of pressure to conform or perceived acceptability) and social learning (adoption of an attitude/behavior based on information acquisition or observation of others’ engagement in this behavior) as the primary mechanisms to consider (Cislaghi & Heise, 2019; Cislaghi & Shakya, 2018). While the focus on individual rights in SRH programs and policies should be paramount, without consideration of the normative environment, it will remain difficult to implement programs in such a way that they facilitate achievement of these individual rights. Utilizing a social network approach has important potential to illuminate the social mechanism(s) through which SRH messages diffuse and ultimately the diffusion mechanisms of individual FP-related behaviors. This assertion and thus, our work is supported by a conceptual framework laid out by Price and Hawkins (2002) who argue that social analysis is needed to offer social and cultural context to reproductive health behaviors. (Price & Hawkins, 2007).

Thus far, social network methods in FP research have been used primarily to examine the impact of women's networks on fertility and reproductive behaviors. A 2014 review by Lowe and Moore highlighted that by and large, social learning is the likely mechanism of women's networks while also recognizing the dearth of research seeking to uncover the influence of men's networks on their FP attitudes and behaviors (Lowe & Moore, 2014). One study in Malawi demonstrated that men's FP behaviors were informed by whether or not they believed that their network members also engaged in these behaviors (Paz Soldan, 2004). Another study in Ghana demonstrated that with encouragement to use FP methods from network members, men were more likely to report spousal communication about FP and subsequently, FP method use (Avogo & Agadjanian, 2008). Results of a study in Kenya suggested that social networks have a more profound effect on individuals' behaviors when an innovation, or new behavior, has not yet been widely adopted amongst members of the network. (Behrman & Watkins, 2002) However, these findings are mixed, particularly with respect to what social mechanisms are prominent in men's networks, highlighting the importance of conducting context-specific social analyses. Specifically, in given the prevalence of early marriage in Niger, increased focus on husbands of adolescent girls is merited, since given that these men's support may be more critical to facilitate desired FP-related behaviors compared to the support of men in relationships with older women. Despite our growing understanding of the role of women's networks, social networks and their influences on the FP decision-making process are likely gender-specific given normative, gender role expectations. So, more research is needed to expound on what role men's networks play in these attitudes and behaviors. Specifically, whether it is simple observation of and learning from others that perpetuates behavior, or whether behavior is driven more by perceived acceptance from or disapproval by network members, continues to be understudied. An improved understanding of this dynamic will likely allow for more nuanced program development that accounts for these social mechanisms.

In seeking to better describe the complex social dynamics that contribute to the high fertility and low FP method use context of Niger where early marriage remains prevalent, a social network study was carried out in parallel to the *Reaching Married Adolescents in Niger* (RMA) Study, an FP program evaluation study. The RMA and social networks studies included adolescent wives (ages 13–19), their husbands, and their important social contacts (alters). The present study aims to explore who may influence men's FP decisions and to generate hypotheses on the social mechanisms influencing these men's FP attitudes and behaviors to better inform programmatic efforts.

## 2 Materials and methods

### 2.1 Reaching Married Adolescents program and study

To address high fertility and low FP method use in Niger, the RMA program was developed and implemented to increase FP method use, create an enabling environment for FP, and improve gender equity among adolescent wives and their husbands in the Dosso region of Niger. The program included gender-segregated household visits by local community health workers to provide knowledge and encourage support for FP, and gender-segregated small group discussions to encourage conversations and social cohesion around SRH topics. To assess the effectiveness of the program in promoting FP method uptake, the RMA study includes a four-arm cluster randomized controlled trial (ClinicalTrials.gov NCT03226730) comparing the effects of household visits, small group discussions, and a combination of the two interventions against a control condition.

Across the Dosso, Doutchi, and Loga districts of the Dosso region, 16 villages per district (48 total) were randomly selected with 12 randomly assigned to treatment condition and 4 to control within each district. Eligible men were married to an adolescent wife between the ages of 13–19. Baseline data were collected in 2016 and follow-up data were collected in 2018. Data were collected by gender-matched research assistants who obtained men's verbal consent and administered the survey orally in a private location of the participants' choice using pre-programmed tablets. Surveys took 45–60 min to complete in either Hausa or Zarma, depending on the participant's preference. More details on the intervention, study design, and data collection protocol can be found in Challa et al. (2019).

### 2.2 Social networks study

At follow-up in 2018, a parallel social network study was carried out with participants in the Dosso district (16 villages – 12 intervention and 4 control). The social network module was added to the original participant survey and included three questions to obtain the names of alters including: 1) Who do you trust to talk to about personal and important matters, 2) With whom do you discuss decisions about family, including decisions around fertility and family planning, and 3) Are there any additional people who help you make decisions about delaying or spacing pregnancy. Using these types of name generator questions is an established procedure in studying social networks through survey data (Marin, 2004; Shakya et al., 2017). For each question, original participants could name up to three alters (up to nine total). Criteria for alters included being over the age of 13 years and residing in the village (so that they could be located for participation in a short alter survey). For each alter nominated who resided in the village, original participants were asked follow-up questions including the alters' place of residence, gender, relationship to the participant, number of children, marital status, age, and participants' perceptions of alters’ gender equity and FP-related attitudes. Original participants were finally asked to rank all nominated alters in order of their level of influence.

Then, for each original participant that nominated alters, one of their alters was approached, recruited, and consented (verbally) for participation. Primary alters (most influential) were approached first followed by secondary alters (second most influential) in cases where the primary alters were unavailable or refused to participate. The alter survey comprised a subset of questions from the original participant survey including those related to attitudes supporting FP and FP behaviors (actual FP method use and spousal communication about FP). It was never disclosed to the interviewed alters who had nominated them. Since their data would have already been recorded, any nominated alter that was an original survey participant had their demographic information noted but was not re-interviewed. The RMA and social networks studies were approved by the Institutional Review Board of the University California San Diego and the Institutional Review Board of the Nigerien Ministry of Health.

### 2.3 Measures

From the original participant survey, we measured men's perception of their alters' attitudes by asking what their alters would think of: 1) a man listening to his wife's fertility preferences – “If a man listens to his wife's preferences around fertility decisions (how many children to have, how far apart they should be, whether or not to use a modern family planning method) [name of alter] will think it is good, bad or neither” and 2) participants' own FP use – “If you decide to use modern family planning to space or limit the number of children you have, [name of alter] will think it is good, bad, or neither”. In addition to good, bad, and neither good nor bad, participants could say they didn't know what their alter would think or decline to respond. To compare agreement with these items against any other response, analyses were completed by excluding ‘decline’ responses and combining ‘neither good nor bad’, and ‘don't know’ responses with ‘bad’. We also included several men's demographic variables that could impact their relationships and have been demonstrated in the literature to be associated with FP-related beliefs and behaviors. These included continuous measures of men's age and their number of children, a categorical measure of their educational attainment (attendance at government school, attendance at Quranic school, or no schooling), and a binary measure of their migration status (whether in the past year they had left their village for a period of three months or more for work).

In both the original participant and alter surveys, original participants and their alters were asked about their attitudes supporting FP. For analysis, this measure comprised three items to which response options were ‘agree’, ‘disagree’, ‘don't know’, or ‘decline’. Again, to capture explicit comparison against agreement with these items, all ‘don't know’ responses were combined with ‘disagree’, and ‘decline’ responses were made missing. Only those with valid observations (non-missing, non-declined) on all items were retained. Responses were summed for a score ranging from 0 to 3, with a higher score representing more supportive FP attitudes. The three items had acceptable reliability (Cronbach's Alpha = 0.64) and included: 1) “It is acceptable for a couple to try to limit the number of children they have”, 2) “It is acceptable for a couple to use a family planning method so they can have fewer children”, and 3) “It is acceptable for a couple to use a family planning method to space or delay pregnancy”. We also studied men's and alters' FP-related behaviors. These included ever use of FP methods, a binary variable capturing whether they had ever done something or used any method to space or delay pregnancy, and ever having spousal communication about FP, a binary variable capturing whether they had ever discussed using a FP method to space or delay pregnancy with their wives.

### 2.4 Analysis

First, we examined the sample descriptively to understand the demographic characteristics of the original participants and both nominated and interviewed alters. We analyzed an *individual dataset* with unique respondent observations that contained original participants' reported attitudes, behaviors, and perceptions. Using this *individual dataset*, we explored what demographic characteristics were linked to the number of alter nominations, as a key descriptor of network size. For analyses with this *individual dataset*, we used logistic regression models adjusting for men's age, education, number of children, migration status, and treatment condition (intervention vs control).

Next, we created dyads consisting of each original participant (known in social networks research as “egos”) and each of the alters that he nominated in the network module, including the egos' survey responses about his own attitudes and behaviors and his own responses specific to his perceptions of each alter nominated. Importantly this dataset did not include any direct survey responses from any of the alters nominated – it was limited solely to egos answers to questions about the alters he nominated. An ego who nominated multiple alters would have separate observations for each alter nominated. Using this *egocentric dataset*, we assessed associations between egos' perceptions of their alters and egos' own FP-related attitudes and behaviors, including actual FP method use and spousal communication about FP. We excluded any alters who lived outside the village (N = 16), as follow-up questions were not asked about those that could not be located. We used generalized estimating equations (GEE), specifying individual-level repeated measures, to account for the possibility of multiple observations for respondents with more than one alter nomination, and an independent correlation structure. These models adjusted for men's age, education, number of children, migration status, number of alters nominated, and treatment condition (intervention vs control).

Finally, we analyzed a *linked, dyadic dataset*, which comprised unique dyads of egos and *their interviewed alters* (by definition, limited to one alter per original participant). This dataset included egos' survey responses alongside alters' own survey responses and allowed for direct assessment of associations between egos' FP-related attitudes and behaviors and alters' own self-reported FP-related attitudes and behaviors. For these analyses, we utilized generalized linear mixed models (GLMM) specifying random intercept models with village-level random effects to account for clustering. We also adjusted for treatment condition. As our goal was to specifically understand egos’ relationship with alters who were men (a vast majority of alters interviewed), and because normative influence likely differs by gender, women alters were excluded (N = 7).

We used *SAS Studio*® (SAS Institute Inc., 2018) for all analyses. Statistical significance was set at p < 0.05 for all comparisons including adjusted odds ratios (AORs); 95% confidence intervals (CIs) are reported throughout.

## 3 Results

### 3.1 Description of the egos and alters

In this sample there were 367 men in the Dosso district, 237 of whom had valid survey data, participated in the social network study and could have nominated alters. Of these men, 206 (87%) nominated at least one alter while 31 (13%) did not nominate anyone (Table 1). In total, there were 342 nominations, an average of 1.4 alter nominations per ego. Excluding those nominated outside the village resulted in an *individual dataset* containing 206 unique egos who had nominated any alters and an *egocentric dataset* with 326 pairs of egos and *nominated* alters. Of the 206 unique alter nominations, 164 alters, who lived the same village as the nominating ego and were ranked as either primary or secondary in order of influence, participated in interviews. After excluding the 7 interviewed alters who were women, there were 157 pairs of egos and *interviewed* alters in the *linked, dyadic dataset*.

**Table 1:** Characteristics of egos and alters – reported by egos.

	Mean	SD	N(%)
Participants (N = 237)			
Age	27.4	5.2	
Number of Children	2.5	2.8	
Education			
No Schooling			48(20.3)
Government			123(51.9)
Quranic			66(27.9)
Ever Used Family Planning Methods (Modern or Not)			97(40.9)
Ever Communicated with Wife about Family Planning			133(56.1)
Migration Status (traveled from village for >3 months in past 12 months)			140(59.1)
Treatment Group			177(74.7)
Alters Nominated	1.4	0.9	
No Nominated Alters			31(13.1)
Alter (N = 326)			
Age (25% don't know)	29.2	8.4	
Number of Children	2.3	2.1	
Relationship with Participant - Female			
Mother			4(1.2)
Sister			3(1.0)
Other Family Member			5(1.5)
Other			8(2.5)
Relationship with Participant - Male			
Friend			219(67.2)
Brother			40(12.3)
Other Family Member			37(11.4)
Other			10(3.1)
Perceived Support for FP method use (10% don't know)			247(75.8)
Perceived Support for Men Listening to Wives' Fertility Preferences (9% don't know)			249(76.4)
Participated in a Survey			164(48.0)

Egos were on average 27 years of age and had, on average, 2.5 children, with 52% having attended government school. Additionally, 41% reported ever having used FP methods while 56% reported ever having spousal communication about FP. When asked about their alters, egos reported that they were on average 29 years of age (though 25% stated they did not know their alters' ages). Of all alter nominations, there were only 20 women, most of whom were family members. Men nominated as alters were mostly friends (67%), brothers (12%), and other family members (11%). A majority of egos perceived their alters to be supportive of both a man listening to his wife's fertility preferences (76%) and supportive of his own FP use (76%).

Alters themselves reported being on average 30 years of age and having 2.9 children (Table 2). Reflective of the alter nominations overall, a majority of alters interviewed were male friends (74%). Half of all alters (49%) had attended government school while 27% reported having no schooling. Additionally, 28% reported ever having used FP methods while 38% reported ever having spousal communication about FP.

**Table 2:** Alter characteristics – reported by alters.

	Mean	SD	N(%)
Alter (N = 157)			
Age	30.4	8.6	
Number of Children	2.9	2.9	
Relationship with Participant			
Friend			116(73.9)
Brother			21(13.4)
Other Family Member			18(11.5)
Education			
No Schooling			43(27.4)
Government			77(49.0)
Quranic			35(22.3)
Ever Used Family Planning Methods (Modern or Not)			44(28.0)
Ever Communicated with Wife about Family Planning			60(38.2)
RMA Participants			27(17.2)

### 3.2 Who nominated alters

In the *individual dataset*, results indicated that no demographic characteristics were associated with nominating at least one alter (Table 3). On the other hand, those who attended government school had greater odds of nominating more than one alter compared to those who attended no school (AOR: 4.98, 95% CI: 1.87, 13.23), as were those who attended Quranic school (AOR: 5.93, 95% CI: 2.07, 17.04). Additionally, men in the treatment group of the RMA Study had higher odds of nominating more than one alter than those in the control group (AOR: 2.16, 95% CI: 1.01, 4.62).

**Table 3:** Associations of demographics and nomination of alters.

	Probability of 1 or More Nominations vs. No Nominations (n = 206, n = 31)			Probability of More than 1 Nomination vs. Only 1 Nomination (n = 107, n = 99)		
	AOR	95% CI	*p*	AOR	95% CI	*p*
Government School vs. No School	1.68	0.50–5.62	*0.40*	**1.98**	**1.87–13.23**	***0.001***
Quranic School vs. No School	2.41	0.57–10.09	*0.23*	**5.93**	**2.07–17.04**	***<0.001***
Number of Children	1.19	0.86–1.64	*0.30*	1.02	0.89–1.16	*0.79*
Age	0.94	0.84–1.05	*0.29*	1.01	0.93–1.07	*0.98*
Migration	0.41	0.13–1.30	*0.13*	0.57	0.28–1.16	*0.012*
Treatment vs. Control	0.47	0.12–1.76	*0.26*	**2.16**	**1.01–4.62**	***0.047***

### 3.3 Egos' perception of alters and associations with egos’ attitudes and behaviors

**Fig. 1:**
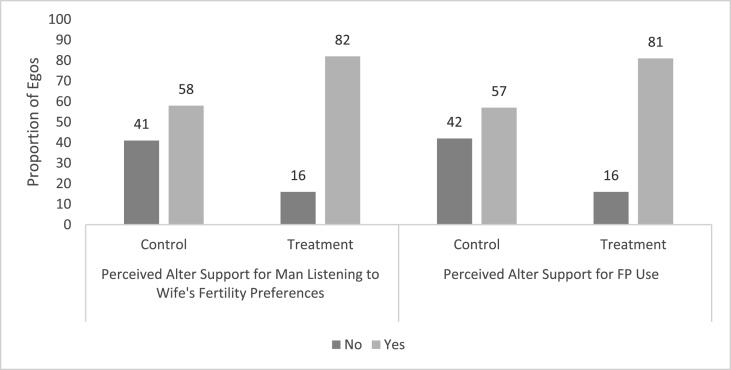
Egos' perceptions of alters' attitudes by treatment condition.

**Fig. 2:**
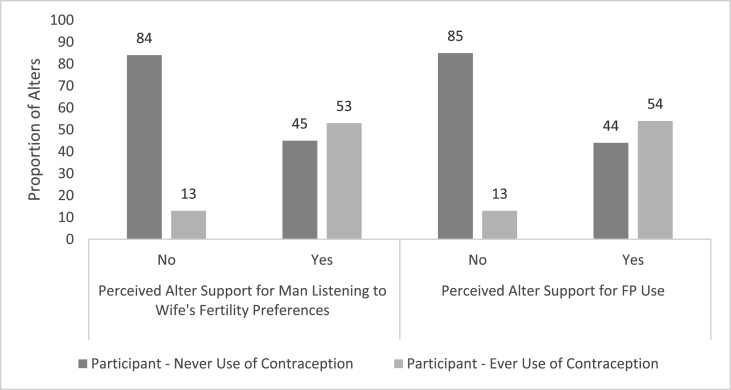
Egos' perceptions of alters' attitudes by contraceptive use status.

**Fig. 3:**
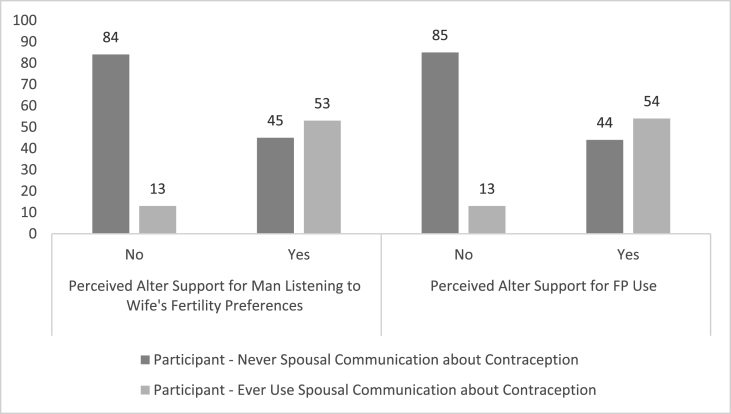
Egos' perceptions of alters' attitudes by spousal communication status.

Using the *egocentric dataset*, which contained pairs of egos and their *nominated* alters, we found that a high proportion of egos in the treatment group perceived alters to support gender equitable FP decisions and the egos' own FP use (Fig. 1). What is more, in regression models, these egos who were RMA intervention participants were more likely to perceive their alters would support a man listening to his wife's fertility preferences (AOR: 4.36, 95% CI: 1.83, 10.35) and to perceive their alters would support the egos' own FP use (AOR: 4.22, 95% CI: 1.72, 10.35) than those in the control group. Additionally, egos who were RMA intervention participants had a higher mean FP attitude score than those in the control group (μ_intervention_ = 2.04 vs (μ_Control_ = 1.89) In regression models, treatment condition was associated with an FP attitude score 0.51 units greater than the control (95% CI: 0.03, 1.00). Egos with attitudes more supportive of FP use were more likely to perceive that their alters support a man listening to his wife's fertility preferences (for each 1-unit increase in attitudes supporting FP, AOR: 2.66, 95% CI: 1.73, 4.08) and to perceive that their alters support the egos' own FP use (for each 1-unit increase in attitudes supporting FP, AOR: 2.79, 95% CI: 1.77, 4.39) (Table 4). Many egos who perceived their alters to support gender equitable FP decisions and their actual FP use, engaged in FP-related behaviors (Fig. 2, Fig. 3). In regression models, egos who believed their alters would support a man listening to his wife's fertility preferences were more likely to have ever used FP methods (AOR: 10.43, 95% CI: 2.50, 43.58) as were those who perceived their alters would support for the egos' own FP use (AOR: 12.76, 95% CI: 2.55, 63.81) (Table 5). Finally, egos who believed alters were supportive of a man listening to his wife's fertility preferences were more likely to report spousal communication (AOR: 8.71, 95% CI: 3.06, 24.83), as were those who perceived that alters would support egos' own FP use (AOR: 9.06, 95% CI: 3.01, 27.26) (Table 6).

**Table 4:** Associations of egos' attitudes supporting FP and perception of alters’ attitudes.

	Perception of Alter Support for Man Listening to Wife's Fertility Preferences			Ego Perception of Alter Support for FP Use		
	AOR	95% CI	*p*	AOR	95% CI	*p*
Attitudes Supporting FP Score (0–3)	**2.66**	**1.73–4.08**	***<0.001***	**2.79**	**1.77–4.39**	***<0.001***
Government School vs No School	1.45	0.31–6.91	*0.64*	1.78	0.36–8.85	*<0.48*
Quranic School vs No School	0.86	0.19–3.96	*0.85*	0.90	0.19–4.33	*0.90*
Number of Children	1.01	0.83–1.21	*0.96*	1.00	0.83–1.21	*0.99*
Age	1.01	0.94–1.09	*0.73*	1.02	0.94–1.10	*0.70*
Migration	0.97	0.38–2.43	*0.94*	0.93	0.36–2.41	*0.87*
Alter Number	**1.92**	**1.06–3.48**	***0.033***	**1.85**	**1.02–3.33**	***0.041***
Treatment	**3.20**	**1.24–8.22**	***0.016***	**3.02**	**1.14–8.00**	***0.026***

**Table 5:** Associations of egos' perception of alters' attitudes and egos’ family planning method use.

	Ego Ever Use of Family Planning Methods						
	AOR	95% CI	*p*		AOR	95% CI	*p*
Ego Perception of Alter Support for Man Listening to Wife's Fertility Preferences	**10.43**	**2.50–43.58**	**0.001**	Ego Perception of Alter Support for FP Use	**12.76**	**2.55–63.81**	**0.002**
Government School vs No School	0.58	0.22–1.53	0.27	Government School vs No School	0.57	0.21–1.50	0.25
Quranic School vs No School	0.44	0.14–1.38	0.16	Quranic School vs No School	0.43	0.13–1.39	0.16
Number of Children	1.15	0.96–1.37	0.12	Number of Children	1.16	0.96–1.39	0.12
Age	1.01	0.93–1.09	0.87	Age	1.01	0.93–1.09	0.88
Migration	0.75	0.34–1.66	0.47	Migration	0.77	0.34–1.73	0.52
Alter Number	1.49	0.84–2.66	0.17	Alter Number	1.48	0.83–2.65	
Treatment	0.63	0.25–1.62	0.34	Treatment	0.64	0.24–1.71	0.38

**Table 6:** Associations of egos' perception of alters' attitudes and egos’ spousal communication about family planning.

	Ego Ever Spousal Communication About Family Planning						
	AOR	95% CI	*p*		AOR	95% CI	*p*
Ego Perception of Alter Support for Man Listening to Wife's Fertility Preferences	**8.71**	**3.06–24.83**	**<0.001**	Ego Perception of Alter Support for FP Use	**9.06**	**3.01–27.26**	**<0.001**
Government School vs No School	2.20	0.80–6.01	0.13	Government School vs No School	2.08	0.76–5.69	0.15
Quranic School vs No School	1.66	0.51–3.43	0.56	Quranic School vs No School	1.63	0.51–5.20	0.41
Number of Children	1.12	0.92–1.35	0.27	Number of Children	1.12	0.92–1.36	0.26
Age	0.97	0.88–1.06	0.49	Age	0.97	0.88–1.06	0.48
Migration	0.50	0.22–1.14	0.10	Migration	0.51	0.22–1.17	0.11
Alter Number	1.12	0.56–2.24	0.75	Alter Number	1.14	0.57–2.28	0.72
Treatment	1.51	0.57–4.00	0.41	Treatment	1.54	0.56–4.25	0.40

### 3.4 Alters' self-reported attitudes and behaviors and egos’ attitudes and behaviors

Using the *linked, dyadic dataset* in which each observation included respondents' reports and their interviewed alters' reports, we found that a 1-unit increase in alters' attitudes supporting FP was associated with a 0.48-unit increase in egos' attitudes supporting FP (95% CI: 0.32, 0.63). Additionally, there was evidence of association between alters' attitudes supporting FP and egos' ever use of FP methods (AOR: 1.31, 95% CI: 0.94, 1.82) and ever having spousal communication about FP (AOR: 1.33, 95% CI: 0.95, 1.86) though these associations did not reach statistical significance (p = 0.11 and p = 0.09, respectively). However, when examining associations of alters' and egos’ FP-related behaviors, neither their use of FP methods nor their reports of spousal communication about FP were found to be associated (Table 7).

**Table 7:** Association of alters' family planning-related behaviors and egos’ family planning-related behaviors.

	Alter FP method Use			Alter Spousal Communication About Family Planning			
	AOR	95% CI	*p*		AOR	95% CI	*p*
Ego FP method Use	1.09	0.52–2.29	0.82	Ego Spousal Communication about Family Planning	1.10	0.51–2.36	0.80
Treatment	2.32	0.96–5.60	0.06	Treatment	**3.12**	**1.09–8.90**	**0.03**

## 4 Discussion

The purpose of this study was to describe the social networks of a sample of men in rural Niger married to adolescent wives. Men in the study were participating in a randomized controlled trial of a program designed to improve gender equity and create an enabling environment for FP use in Nigerien communities. A primary research question in the current study was how perceived norms and network members' FP-related attitudes and behaviors were associated with those of the respondents who nominated them. Our results showed that, along multiple dimensions, there were relevant and statistically significant associations between the attitudes and behaviors of male egos (original study participants) with both their perceptions of their alters' attitudes and behaviors, and with alters’ own self-reported attitudes and behaviors.

Our data showed that most men in the sample named one alter who was influential in personal matters or FP decisions. These alters were primarily friends who were men and close in age to them. In comparison, results from a recent study of the adolescent wives of the men in our sample by Shakya et al. (2020), demonstrated women nominated a similar number of alters to their husbands, but more commonly nominated women relatives, including sisters, other members of their natal family, and in-laws (Shakya et al., 2020). When considering the broader literature, it is relevant to highlight a recent study with men's networks in Benin which similarly found that men named a mean of only two social contacts who were primarily not male relatives (LeMasters et al., 2021). However, several studies with women have found network sizes to be larger and that within these networks, explicit discussions about FP decisions and behaviors occur. (Behrman & Watkins, 2002; Madhavan et al., 2003) These mixed results demonstrate the network composition and dynamics are highly context-specific but, given that the approach to name generator questions is varied across studies and that network analysis in Niger is nascent, more work is needed to elucidate the full networks of men. Thus, cognitive testing may help improve our methods for eliciting responses to these questions allowing for more comparative results across contexts. It may also be pertinent to conduct further qualitative research to understand the content, context, and bounds of FP communication both between married couples and within social networks more broadly.

Three main findings came from the *egocentric dataset*, which contains respondents' reports on all nominated alters. First, treatment condition was associated with greater odds of men perceiving alters to support a man listening to his wife's fertility preferences and to support egos' own FP use. The RMA program was aimed at improving support for gender equity and for FP, so men who participated and experienced shifts in their attitudes may have desired relationships with alters who held what they viewed as positive attitudes around gender equity and FP. Research has shown that homophily, or the desire to form ties with those with similar characteristics, is a mainstay of many social networks, and has been observed to be a factor in network influence on FP method use (Akinyemi et al., 2019; Lowe & Moore, 2014; Marsden, 1988; McPherson et al., 2001). Additionally, some selectivity may be strategic or influenced by a person's desire to choose network members they can learn or benefit from. Thus, post participation in the program, believing in the positive aspects of gender equity and FP, egos may have sought to form relationships with people they believed supported these attitudes. Alternatively, it is possible that egos' social ties did not substantively change, but that the treatment condition changed egos' *perceptions* of their alters attitudes, reflecting a desire to ascribe positive traits to members of their networks. These findings do suggest that FP programs, while aiming to change individual attitudes and behaviors, may impact network perception or composition of networks. This finding offers some preliminary evidence that perceived approval is an important determinant of men's attitudes and behavior and that social influence may be at play. However, given the cross-sectional nature of these data, more work would be needed to ascertain whether and how men are choosing to select network members or modify network composition so that network members' attitudes and beliefs align with their own.

Second, egos' higher FP-related attitudes score (e.g., attitudes more supportive of FP use) was associated with an increased likelihood of believing that their alters support the ego's own FP use. This may indicate that men who have positive attitudes towards FP use either choose alters that also support FP use, or that their perceptions of alters' support of FP was informed by their own beliefs. This again may relate to the attitudinal shifts from the program such that men perceived their alters differently after participation as evidenced by the higher mean attitudes score among intervention participants. The third and final major finding from this data set was that for all egos who nominated alters, egos' perceptions of their alters' approval (of both gender equitable FP decision-making and egos' own FP use) were associated with higher odds of egos reporting both FP method use and couple communication about FP. This suggests that even if men's program participation played a role in the formation of their own attitudes and their selection of network members, men likely value alters' support of their own behaviors.

Results in the *linked, dyadic dataset* (the dataset with ego-interviewed alter dyads), namely the lack of association between egos' and alters' FP-related behaviors (FP method use and spousal communication), may demonstrate the possible boundaries of men's discussions about sensitive topics such as fertility with network members. Research has shown that men, as compared to women, have more indirect discussions with their network members which, in the context of fertility and FP, may mean general discussions about the utility of FP for the safety and health of their families (Paz Soldan, 2004). Through these more indirect or general conversations on fertility topics, men may have inferred their network members' support for FP and sought to hold attitudes or engage in behaviors these social contacts would deem appropriate. However, because of a lack of more explicit discussions, egos did not know of alters' actual behaviors. This may be why present findings indicate alters' attitudes were related to egos' attitudes and behaviors, but alters' behaviors were not related to those of the ego. The limited nature of discussion around sensitive topics among men and their social contacts can be compared to findings from social networks analysis with women which suggests women engage in much more detailed discussions within their networks including on topics such as FP method methods used, side effects, etc. (Paz Soldan, 2004; Rutenberg & Watkins, 1997). Again, as shown by Shakya et al. (2020), the adolescent wives in this sample did report FP method use that was found to be associated with their alters' FP use, with these associations varying based on the wives' relationship to their alters (Shakya et al., 2020).

Understandably, the historical focus of FP promotion programs on women has spurred interest in studying the influence of women's social networks. However, with increased male involvement in these programs, and the apparent differences between women's and men's social relationships, specific attention should be paid to the study of men's networks, particularly considering their decision-making power in this, and many other, contexts. Taken together, the findings of this study highlight the importance of men's perceptions of their alters' attitudes and the alters' own self-reported attitudes. The focus on attitudes may suggest the importance of perceived approval and thus, a pattern of social influence in men's networks. This social conformity pressure has been shown to be a characteristic of denser, more isolated, and more homogenous networks, like the rural communities that participated in this study, and since acting outside normative behavior may mean facing harsh repercussions, norms in such networks may be very difficult to change (Kohler HP et al., 2001). In this case, programs that motivate early adoption of new beliefs or behaviors (such as those related to FP) that break from the norm, may result in the imposition of social sanctions thereby causing a disruption in networks.

Thus, if programs focused on facilitating positive attitudes around FP and actual FP use operate on only an individual level, unintended consequences may include breaking of social ties with those who are early adopters. While we would need longitudinal data to truly understand the social mechanism in effect, our findings provide evidence that programs that engage broader networks of men may have more success due to wider acceptance of FP-related attitudes and behaviors. Per a recent meta-analysis of network interventions in health research, these efforts may take the form of individual interventions to identify specific individuals as proponents of behavior change, segmentation interventions that direct efforts to specific network clusters, induction interventions that activate existing ties to accelerate diffusion, or alteration interventions that aim to change the structure of networks to impact behavior (Hunter et al., 2019). However, given the contextual nature of networks, it is critical to understand context-specific structures and dynamics, evidence we have sought to produce through this study.

Our results should be considered with several limitations in mind. First, the sample size of this study is quite small, precluding our ability to test more complex models and limiting the strength of the conclusions that can be drawn. Second, cross-sectional data prevent us from establishing temporality and from causal interpretation of findings, since we don't have information about the networks and related norms and behaviors at baseline prior to program implementation. Additionally, these data were self-report and topics related to SRH, and FP are considered sensitive in rural Nigerien communities, introducing the possibility of social desirability bias. Finally, since the social networks module was administered at follow-up, there is some chance of response bias due to RMA participants understanding the aim and intention of the program and study. However, control egos were also included and alters interviewed were primarily non-participants, so risk of this response bias would have been limited. Future research on men's social networks should include larger samples and longitudinal data to strengthen understanding of the structure and formation of network relationships and to assess how observations of others' FP behaviors shape men's decisions. This understanding may be particularly important in the context of programs that take social networks approach to influence social norms and health behaviors.

## 5 Conclusions

Our findings provide new and important insight into Nigerien men's social dynamics around fertility and FP decisions, demonstrating that in addition to women, men may in fact be influenced by their network members. Critically, social influence may be the driving force behind this influence on men's FP-related attitudes and behaviors in this context. Future research with larger sample sizes and longitudinal data will help to understand these networks more clearly, including clarifying how they are formed and modified. As men are often decision-makers around FP in the Nigerien context, with a better understanding of the operational social mechanisms, we will be better equipped to determine whether individual, segmentation, induction, or alteration approaches would be appropriate to facilitate FP method use among those who desire it. In this environment of high fertility and low FP method use, promoting the acceptability of FP-related behaviors through social networks may enhance the process of social change around timing and spacing of pregnancy, ultimately improving the support for young married women's FP method choices.

## Ethical statement

All consent and data collection procedures were approved by the Institutional Review Board of the University of California San Diego (ID: 1604075, August 03, 2016) and the Ethics Committee of the Ministry of Health of Niger.

## Author statement

**Sneha Challa:** Conceptualization, Formal Analysis, Writing – Original Draft, Project Administration.

**Nicole Johns:** Formal Analysis, Writing – Review & Editing, Project Administration.

**Abdoul Moumouni Nouhou:** Investigation, Project Administration.

**Ricardo Vera-Monroy:** Data Curation, Project Administration.

**Jay G Silverman:** Supervision, Writing – Review & Editing, Funding Acquisition.

**Holly Shakya:** Conceptualization, Writing – Review & Editing, Funding Acquisition.

## Funding

This work was supported by the Bill and Melinda Gates Foundation [OPP1195210, Prime: Pathfinder International; Research PI: J Silverman]. The Bill and Melinda Gates Foundation did not have any additional role in the study design, data collection and analysis, decision to publish, or preparation of the manuscript.

## Declaration of competing interest

None.

## References

[bib1] Akinyemi O., Harris B., Kawonga M. (2019). Innovation diffusion: How homogenous networks influence the uptake of community-based injectable contraceptives. BMC Public Health.

[bib3] Avogo W., Agadjanian V. (2008). Men's social networks and contraception in Ghana. Journal of Biosocial Science.

[bib4] Behrman K.H., Watkins S.C. (2002). Social networks and changes in contraceptive use over time: Evidence from a longitudinal study in rural Kenya. Demography.

[bib28] Challa Sneh, DeLong Stephanie, Carter Nicole, Johns Nicole, Shakya Holly, Boyce Sabrina C., Silverman Jay G. (2019). Protocol for cluster randomized evaluation of reaching married adolescents - a gender-synchronized intervention to increase modern contraceptive use among married adolescent girls and young women and their husbands in Niger. Reproductive Health.

[bib6] Cislaghi B., Heise L. (2019). Using social norms theory for health promotion in low-income countries. Health Promotion International.

[bib7] Cislaghi B., Shakya H. (2018). Social norms and adolescents' sexual health: An introduction for practitioners working in low and mid-income African countries. African Journal of Reproductive Health.

[bib8] Family Planning 2020 (2018).

[bib9] Green L.W., Richard L., Potvin L. (1996). Ecological foundations of health promotion. American Journal of Health Promotion.

[bib10] Kohler H.P., Behrman J.R., Watkins S.C. (2001). The density of social networks and fertility decisions: Evidence from South Nyanza District, Kenya. Demography.

[bib11] Lowe S.M.P., Moore S. (2014). Social networks and female reproductive choices in the developing world: A systematized review. Reproductive Health.

[bib12] Luke D.A. (2005). Getting the big picture in community science: Methods that capture context. American Journal of Community Psychology.

[bib13] Luke D.A., Harris J.K. (2007). Network analysis in public health: History, methods, and applications. Annual Review of Public Health.

[bib27] Madhavan S., Adams A., Simon D. (2003). Women’s Networks and the social world of fertility behavior. Int Fam Plan Perspect.

[bib14] Marin A. (2004). Are respondents more likely to list alters with certain characteristics? Implications for name generator data. Social Networks.

[bib15] Marsden P.V. (1988). Homogeneity in confiding relationships. Social Networks.

[bib16] Masquelier A. (2005). The scorpion's sting: Youth, marriage and the struggle for social maturity in Niger. The Journal of the Royal Anthropological Institute.

[bib17] McPherson M., Smith-Lovin L., Cook J.M. (2001). Birds of a feather: Homophily in social networks. Annual Review of Sociology.

[bib18] Paz Soldan V.A. (2004). How family planning ideas are spread within social groups in rural Malawi. Studies in Family Planning.

[bib19] Perlman D., Chaibou S. (2018).

[bib21] Rutenberg N., Watkins S.C. (1997). The buzz outside the clinics: Conversations and contraception in Nyanza province, Kenya. Studies in Family Planning.

[bib22] Samandari G., Grant C., Brent L., Gullo S. (2019). It is a thing that depends on God": Barriers to delaying first birth and pursuing alternative futures among newly married adolescent girls in Niger. Reproductive Health.

[bib2] Shakya H.B., Challa S., Nouhou A.M., Vera-Monroy R., Carter N., Silverman J. (2020). Social network and social normative characteristics of married female adolescents in Dosso, Niger: Associations with modern contraceptive use.

[bib23] Shakya H.B., Christakis N.A., Fowler J.H. (2017). An exploratory comparison of name generator content: Data from rural India. Social Networks.

[bib24] United Nations (2015).

[bib25] United Nations Children’s Fund (2017).

[bib26] United Nations Population Fund (US) (2011). https://www.unfpa.org/news/'school-husbands'-encourages-nigerien-men-improve-health-their-families.

